# Age-Related Shifts in Theta Oscillatory Activity During Audio-Visual Integration Regardless of Visual Attentional Load

**DOI:** 10.3389/fnagi.2020.571950

**Published:** 2020-09-30

**Authors:** Yanna Ren, Shengnan Li, Tao Wang, Weiping Yang

**Affiliations:** ^1^Department of Psychology, College of Humanities and Management, Guizhou University of Traditional Chinese Medicine, Guiyang, China; ^2^Department of Psychology, Faculty of Education, Hubei University, Wuhan, China; ^3^Department of Light and Chemical Engineering, Guizhou Light Industry Technical College, Guiyang, China

**Keywords:** audio-visual integration, attentional load, theta-band oscillation, alpha-band oscillation, race model, older adult

## Abstract

Audio-visual integration (AVI) is higher in attended conditions than in unattended conditions. Here, we explore the AVI effect when the attentional recourse is competed by additional visual distractors, and its aging effect using single- and dual-tasks. The results showed the highest AVI effect under single-task-attentional-load condition than under no- and dual-task-attentional-load conditions (all *P* < 0.05) in both older and younger groups, but the AVI effect was weaker and delayed for older adults compared to younger adults for all attentional-load conditions (all *P* < 0.05). The non-phase-locked oscillation for AVI analysis illustrated the highest theta and alpha oscillatory activity for single-task-attentional-load condition than for no- and dual-task-attentional-load conditions, and the AVI oscillatory activity mainly occurred in the Cz, CP1 and Oz of older adults but in the Fz, FC1, and Cz of younger adults. The AVI effect was significantly negatively correlated with FC1 (*r*^2^ = 0.1468, *P* = 0.05) and Cz (*r^2^* = 0.1447, *P* = 0.048) theta activity and with Fz (*r*^2^ = 0.1557, *P* = 0.043), FC1 (*r*^2^ = 0.1042, *P* = 0.008), and Cz (*r*^2^ = 0.0897, *P* = 0.010) alpha activity for older adults but not for younger adults in dual task. These results suggested a reduction in AVI ability for peripheral stimuli and a shift in AVI oscillation from anterior to posterior regions in older adults as an adaptive mechanism.

## Introduction

Individuals are often inundated with stimuli from various sensory modalities (e.g., auditory, visual, olfactory, and somatosensory stimuli) (Xu et al., 2020). For example, when a train is approaching, there are noisy engine sound, mobile train, flowed air, raised dust, which we percept from different senses. But the individual knows it is a speeding train, and is even able to estimate its speed. The process merging information from different modalities is called multisensory integration (Spence, 2011; Stein, 2012). Most people perceive the outside world by relying more on visual information and auditory information, and the procedure merging information from auditory and visual modalities is called audio-visual integration (AVI) (Meredith et al., 1987; Stein and Meredith, 1993; Laurienti et al., 2006). The AVI assists us to accurately perceive the outside world, and these studies confirmed that the response to bimodal audiovisual stimulus was faster than to unimodal auditory or visual stimulus (Meredith et al., 1987; Stein and Meredith, 1993; Stein, 2012). Attention is also a key factor that alters the sensory processing by enhancing the perception of the attended location (Mcdonald et al., 2000; Ho et al., 2009; Xu et al., 2020). Both AVI and attention are able to aid in the stimuli detecting, discriminating, and locating, and thus assisting us in effectively recognizing the outside world (Stein and Stanford, 2008; Spence, 2010; Xu et al., 2020).

Talsma and Woldorff (2005) and Talsma et al.(2007, 2009, 2010) reported that attention affects AVI in numerous stages and that the AVI effect is higher in attended location than unattended location. According to the attentional load theory proposed by Lavie and Tsal (1994) people have limited attentional resources; when multiple tasks are conducted simultaneously, if one task uses more attentional resources, less attention will be allocated to the other tasks. In addition, when humans face more than one event, the main task is often disturbed by distractors from the environment (Fan et al., 2002). Alsius et al. (2005) evaluated the interaction between attentional load and AVI through examining participant’s susceptibility using the McGurk illusion task in dual- and single-task conditions. In the dual task, the McGurk words and a distractor were presented at the same time, and participants were instructed to repeat the McGurk words and press a button every time they saw or heard repetitive distractor stimuli. However, the participants were asked only to repeat the McGurk words and to try their best to ignore the distractor in the single task. The results illustrated that susceptibility to the McGurk illusion was lower in the dual-task condition comparing that in the single-task condition, which suggested that the AVI effect was weakened by concurrent additional distractors resulting from diverting attentional resources from the McGurk illusion task. To further demonstrate whether the decreased AVI was caused by the competition of attentional resources form the additional distractor, Alsius et al. (2007) examined the AVI when attentional recourse was occupied by touch stimuli that did not include auditory or visual perception using in the McGurk illusion task. The results were consistent with their previous study, showing that AVI was reduced even though that attentional recourse was diverted to the irrelevant touch stimuli. Alsius et al. (2014)’s subsequent electroencephalogram study illustrated that N1 and P2, the early auditory event-related potentials (ERP) components, peaked earlier in response to audio-visual (AV) stimuli than in response to auditory stimuli in a single task, but the latency decrement was reduced when attention was loaded in a dual task. This suggested that the AVI effect was weakened in the attentional load condition.

Attentional load theory proposes that if all tasks are simple enough, they can be completed successfully. However, if the tasks are difficult, the main task can be completed by being allocated more attentional resources, but the other tasks cannot be completed because of attentional resource exhaustion. In the studies by Alsius et al., speech perceptual paradigm was used, and the attentional load was too high, with an accuracy lower than 60% in dual tasks (Alsius et al., 2005, 2007, 2014). Using simple AV stimuli, dynamic hand-held tool stimuli, and speech stimuli, Stevenson et al. assessed the binding window of AVI, which is an important index for when AVI occurs (Stevenson and Wallace, 2013). Their results showed a wider binding window for speech stimuli than for non-speech stimuli, which indicated that the experimental material greatly affected AVI. High-cognitive-demand speech-integration materials were used in the studies by Alsius et al., however, it remained unclear whether attentional load influences the AVI during simple stimuli processing under relatively weak attentional load. Therefore, currently, a particular interest was to investigate the AVI effect when the attentional recourse was competed by additional distractors during simple audio-visual processing. Because the single-task and dual-task were supported by distinct neural mechanisms (Worringer et al., 2019), we hypothesized that the attentional load affected AVI regardless of attentional-load conditions, but modulation of attentional load on AVI effect was different when the attentional load was from single- and dual-task. In order to test this hypothesis, an AV discrimination task, for evaluating the AVI effect, was applied individually or accompanied by a simultaneous rapid serial visual presentation task (RSVP), for manipulating attentional load by competing attentional recourse with AV discrimination task (Ho et al., 2009). If the AVI effect was different between single-task-with- and without-distractor conditions, and between single- and dual-task conditions, it will be possible to conclude that attentional load influence AVI effect differently in single- and dual-tasks.

Additionally, visual acuity tends to decrease and the auditory threshold tends to increase with age (Laurienti et al., 2006; Diederich et al., 2008), and this deterioration is able to lead to poorer health status and cognitive functional decline in older adults (Freiherr et al., 2013). However, studies have reported a higher AVI effect for older adults than for younger adults in AV discrimination tasks (Peiffer et al., 2007; Diederich et al., 2008; Zou et al., 2017), sound-induced flash illusion tasks (Deloss et al., 2013), semantic discrimination tasks (Laurienti et al., 2006), and speech perception tasks (Sekiyama et al., 2014). Researchers have further proposed that enhanced AVI might compensate for the unisensory functional decline by enhancing the activity in original brain regions (Diaconescu et al., 2013; Zou et al., 2017), recruiting additional brain areas (Diaconescu et al., 2013; Wang et al., 2018) or strengthening brain functional connectivity (Wang et al., 2018) in the completion of some cognitive tasks. Aging-related studies have shown that there are distractor-suppression deficits in older individuals (Plude et al., 1994; Kok, 2000; Quigley et al., 2010). Older adults were found to be much more susceptible to irrelevant distractors, showing a significant performance reduction compared to younger adults with the addition of distractors (Williams et al., 2016). However, the way attentional load affects age-related AVI has not yet been studied. Therefore, another interest of the current study was to investigate whether there was significant diversity between older and younger adults when participants’ attention resources were reduced resulting from the occupation by additional distractors of the RSVP task. Considering that the older adults can still correctly percept the outside world, we hypothesized that the aging brain undergoes adaptive changes, and the hypothesis was tested by comparing the AVI effect and its brain regions between older and younger adults under all attentional load conditions. If the AVI effect was higher for older adults than for younger adults, or the AVI-relevant brain region is different between older and younger adults, it will be possible to conclude the existence of adaptive mechanism for older adults during integration of auditory and visual stimuli.

Neural oscillations is linked to various perceptual and cognitive brain operations and could be a preferred way to track the activities of neurons (Senkowski et al., 2007). Oscillation activities could provide key physiological information on brain dynamics, and is an efficient method to investigate the time course of AVI. In addition, previous studies have shown that neural oscillatory responses in the theta (4–7 Hz) and alpha (8–13 Hz) bands provide a potential mechanism for cross-modal integration and information selection (Senkowski et al., 2008). Theta oscillation has been suggested to be associated with attention arousal (Keller et al., 2017), and alpha-band activity has been recognized as a marker of when stimuli are being intentionally ignored (Yordanova et al., 1998; Friese et al., 2016; Keller et al., 2017). Therefore, in the present study, theta and alpha oscillatory activity were monitored to evaluate the AVI effect for different attentional loads.

## Materials and Methods

### Participants

Twenty healthy older adults (57–70 years old, mean age ± *SD*, 63.30 ± 3.01) and 20 healthy younger adults (19–24 years old, mean age ± *SD*, 21.00 ± 1.56) were recruited to participate in the study. All participants were paid 60 RMB per hour for their time and completed the experiment successfully. All of the younger adults were college students at Hubei University, and the older adults were citizens of Wuhan City. All participants were free of neurological diseases, had normal or corrected-to-normal vision, have no color blindness, color weakness, or hearing threshold, and were naïve to the purpose of the experiment. Participants were excluded if their Mini-Mental State Examination (MMSE) scores were greater than 2.5 SDs from the mean for their age and education level (Bravo and Hébert, 1997). Additionally, participants who reported a history of cognitive disorder were excluded from the experiment. All participants provided written informed consent for the procedure, which was previously approved by the Ethics Committee of Hubei University (No. 2019106) and the Second Affiliated Hospital of Guizhou University of Traditional Chinese Medicine (No. 2018072).

### Stimuli

**FIGURE 1:**
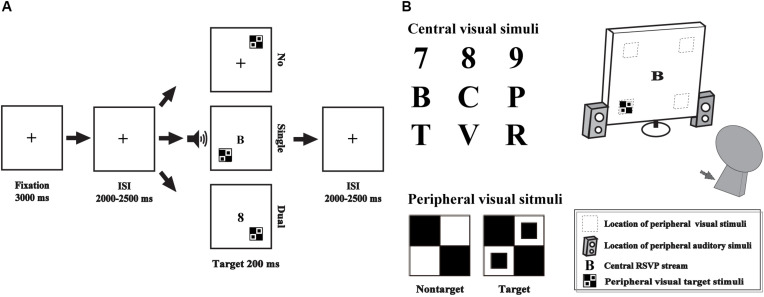
Schematic depiction of the experimental design **(A)**. Audio-visual integration was evaluated by an audio-visual discrimination task, which was presented peripherally (gray square and speakers), and attentional load was manipulated using the RSVP task, which was presented centrally **(B)**. No, no-attentional-load condition; single, single-task-attentional-load condition; Dual, dual-attentional-load condition.

The single- and dual-tasks were performed in the current study, including an AV discrimination task for evaluating the AVI effect and a RSVP task for manipulating attentional load by competing attentional recourse with AV discrimination task as the additional distractors (Ho et al., 2009). The AV discrimination task and RSVP task were presented simultaneously or not simultaneously, according to the attentional load session (Figure 1).

For the AV discrimination task, the visual non-target stimulus was a black and white checkerboard image (B/W checkerboard, 52 × 52 mm, with a visual angle of 5°), and the visual target stimulus was a B/W checkerboard image with two black dots contained within each white checkerboard (He et al., 1996; Talsma and Woldorff, 2005; Ren et al., 2016). The auditory non-target stimulus was a 1000 Hz sinusoidal tone, and the auditory target stimulus was white noise (Yang et al., 2015; Ren et al., 2016, 2018b). The AV target stimulus was the combination of visual target and auditory target stimuli, and the AV non-target stimulus was the combination of visual non-target and auditory non-target stimuli. The following conditions were not included: a visual target stimulus accompanied by an auditory non-target stimulus and a visual non-target stimulus accompanied by an auditory target stimulus. The visual stimuli (V) were presented on a computer monitor in front of participants’ eyes and on the upper/lower left or right quadrant of the screen for 200 ms with a 12-degree visual angle (Figure 1B, gray square). The auditory stimuli (A) were presented through two speakers at approximately 60 dB SPL for a duration of 200 ms (10 ms of the rise/fall cosine gate) (Ho et al., 2009; Ren et al., 2016).

The central visual stimuli in the RSVP task consisted of 10 distractor characters taken from 6 letters (B, C, P, R, T, V) and 4 digits (6, 7, 8, 9) presented on a blank screen (52 × 52 mm in size) at the center of the screen (Figure 1B).

### Procedure

Subjects were instructed to perform the experiment in a dimly lit, electrically shielded and sound-attenuated room (laboratory room, Hubei University, China) with their head positioned on a chin rest. Three separate sessions were conducted, including no-attentional-load session, a single task without distractors; single-task-attentional-load session, a single-task with simultaneous visual distractors from RVSP task; and dual-task-attentional-load session, simultaneous presentation and response for AV discrimination task and RVSP task.

In the no-attentional-load session, the peripheral AV discrimination task was presented with a “ + ” at the center of the screen (Figure 1A No_load). A fixation cross was presented for 3000 ms, and then, the peripheral A, V and AV stimuli were presented randomly with the inter stimulus interval (ISI) for 2000–2500 ms. The participants were instructed to press the left button of the mouse to respond to the target stimuli as rapidly and as accurately as possible. There is no additional task to compete the attentional recourse with AV discrimination task, so it was called “no attentional load.” In total, there were 20 trials for each target stimulus type (A, V, AV) and 80 trials for each non-target stimulus type (A, V, AV) with an appropriate break for rest according to the specific situation of each subject. To add the attentional load for the AV discrimination task, in the single-task-attentional-load session, when the AV discrimination task was presented identical to that in the no-attentional-load session, the RSVP distractors were presented on the center of the screen simultaneously to compete the attentional recourse with AV discrimination task (Figure 1A single-task-attentional-load, Figure 1B). However, the participant was instructed to only respond to the target stimuli of AV discrimination task while ignoring the central distractors of RSVP stream. Although the participant was instructed to ignore the distractors, it is impossible to disregarding them completely, and it would occupy individual’s attention to some degree, it was called “single-task-attentional-load session” (Alsius et al., 2005; Alsius et al., 2014). In the dual-task-attentional-load session, the AV discrimination task and the RSVP task were presented simultaneously identical to that in the single-task-attentional-load. However, to increase the attentional load for the AV discrimination task, the participant was instructed to response to the target stimuli of AV discrimination task by pressing the left button of the mouse, and to respond to the digits (6, 7, 8, 9) of RSVP task by pressing the right button of the mouse (Figure 1A dual-task-attentional-load). To obtain sufficient trials with no response for EEG analysis, in the dual-task-attentional-load session, 30 trials for each peripheral target stimulus type (A, V, AV) and 120 trials for each peripheral non-target stimulus type (A, V, AV) were conducted, accompanied by a random RSVP letter. The participant was asked to treat the peripheral AV discrimination task and central RSVP task equally. The three sessions were conducted in a random order.

### Data Collection

The behavioral and EEG data were recorded simultaneously. The stimulus presentation and behavioral responses were controlled using E-prime 2.0 (Psychology Software Tools, Inc., Pittsburgh, PA, United States). An EEG system (BrainAmp MR plus, Gilching, Germany) was used to record EEG signals through 32 electrodes mounted on an electrode cap (Easy-cap, Herrsching-Breitbrunn, Germany). Vertical eye movements and eye blinks were detected by acquiring EOG data from an electrode placed ∼1 cm below the subject’s left eye (VEOG). Horizontal eye movements were measured by acquiring an EOG signal from one electrode placed ∼1 cm from the outer canthi of the left eye (HEOG). The impedance was maintained below 5 kΩ. The raw signals were digitized using a sample frequency of 250 Hz, and all data were stored digitally for off-line analysis.

### Data Analysis

#### Behavioral Data

The hit rate is the percentage of correct responses (the response time falls within the average time period ± 2.5 SD) relative to the total number of target stimuli. The hit rates and response times (RTs) were computed separately for each subject for each session and then submitted to a 2_group_ (Older, Younger) × 3_Load_ (No attentional load, Single-task attentional load, Dual -task attentional load) × 3_stimulus_ (A, V, AV) ANOVA (Greenhouse-Geisser corrections with corrected degrees of freedom). The statistical significance level was set at *P* ≤ 0.05, and the effect size (η*_*P*_*^2^) estimates were reported. Identical to our previous studies (Ren et al., 2016, 2018a), the AVI effect was assessed using the race model behaviorally. The independent race model is a statistical prediction model based on the cumulative distribution functions (CDFs) of the summed probabilities of the visual and auditory responses to independent unimodal visual and auditory stimuli (Miller, 1982, 1986). This model allows the direct comparison of the multisensory condition probability to the predicted probability of the unimodal conditions [P(V) + P(A)]-P(V) × P(A)] by segmenting the subject-specific CDFs for each condition using 10-ms time bins. P(V) is the probability of responding within a given timeframe in a unimodal visual trial, and P(A) is the probability of responding within a given timeframe in a unimodal auditory trial. If the probability of the response to an AV stimulus is significantly greater than that predicted by the race model *(t-test, P* ≤ 0.05), integration of the auditory and visual inputs is considered to have occurred. The redundant nature of the bimodal AV conditions was defined by subtracting a subject’s race model CDFs from his/her AV CDFs in each 10-ms time bin to generate a difference curve for each subject. The time window of AVI is the time interval for the occurrence of AVI (Diederich et al., 2008; Ren et al., 2016). The greatest facilitation is defined as the peak benefit and was used to evaluate AVI ability behaviorally, and the duration from the presentation of the target to the peak benefit was defined as the peak latency, which was used to evaluate the time for AVI together with the time window (Ren et al., 2016; Xu et al., 2020).

#### EEG Data Analysis

##### Pre-processing

The EEG data were imported and processed with MATLAB R2013b (MathWorks, Inc., Natick, MA, United States) with the open source EEGLAB toolboxes^1^ (Swartz Center for Computational Neuroscience, La Jolla, CA, United States). The EEG data were positioned according to the 32-channel montage of the international 10/20 system, and only data elicited by the non-target stimuli without any response were analyzed to remove the effect of motor response and decision making. First, the two electrodes monitoring eye movement (HEOG and VEOG) were deleted, and then, the data were re-referenced to the bilateral mastoid electrodes (TP9 and TP10). The remaining continuous EEG data were bandpass filtered from 1 to 40 Hz during recordings at a sampling rate of 250 Hz. The data were divided into epochs with 700 time points (800 ms pre-stimulus and 2000 ms post-stimulus points), and then, an independent component analysis (ICA) was used to remove artifacts from the data, including eye artifacts, frequency interference, muscle artifacts, head movement, and electrocardiographic activity (Makeig et al., 1997; Jung et al., 2001; Delorme and Makeig, 2004). Finally, baseline corrections were made based on the 800 ms to 0 ms pre-stimulus interval data from the ICA-corrected data.

##### Time-Frequency Analysis

Time-frequency representations were performed using a short-time Fourier transform (STFT), applied in 1 Hz steps from 1 to 20 Hz with a hamming window of 200 ms. According to the previous references and the analysis results obtained in our lab, such a time-frequency analysis was chosen to achieve a good trade-off between the time resolution and the frequency resolution in the range of theta- and alpha-band EEG frequencies (1–20 Hz) (Zhang et al., 2012, 2013; Ren et al., 2020). Then, baseline corrections were made based on the 600 ms to 200 ms pre-stimulus interval data. To investigate the existence of AVI, the transformed oscillatory response to AV trials was compared with the transformed oscillatory response of summed A-only trial and V-only trial (A + V) (Senkowski et al., 2005). If more than 24 ms consecutive data points met the alpha criterion of being < 0.05 (6 data points = 24 ms at a 250 Hz digitization rate), the AVI was assumed to be occurred. To evaluate the diversity of the AVI oscillatory activity between each attentional load, electrode, and group, the data in the time course across the significant difference was averaged and submitted to group × attentional load × electrode ANOVA (Greenhouse-Geisser corrections with corrected degrees of freedom). The statistical significance level was set at *P* ≤ 0.05. The SPSS version 16.0 software package (SPSS, Tokyo, Japan) was used for all statistical analyses.

#### Correlation Analysis

To further investigate the relationship between neuronal oscillatory activity and behavioral AVI effect, correlation analysis was conducted (*Pearson’s correlation*, *two-tailed*). Because the AVI effect calculated based on the race model was statistical facilitation cross all participants (Miller, 1982, 1986), and it is unable to calculate the AVI effect of individual participant. Therefore, the multisensory response enhancement (MRE) here was introduced through the index (ii) to evaluate the AVI effect of each individual, and further to study the relationship between the AVI effect and theta and alpha activity (Stevenson et al., 2014; Ren et al., 2018a). The MRE is the response gain attributable to having information from a second sensory modality available (Stein and Meredith, 1993). The variables AV, V, and A represent the mean RTs to each stimulus. All data were subjected to bivariate correlation analysis.

ii=min⁢(A;⁢V)-AV

## Results

### RTs and Hit Rates

**FIGURE 2:**
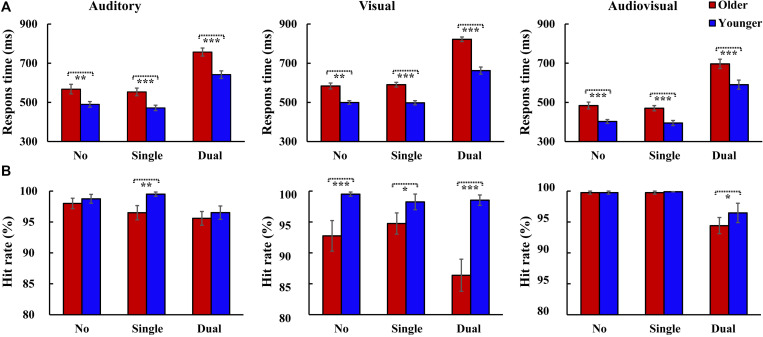
Mean response time and hit rate for 20 participants with the standard errors of the mean (SEM) for each condition. Two-tailed *t*-test showed significantly higher response times were observed for older adults than that for younger adults in all conditions **(A)**, however, the hit rate was relatively lower for older adults than that for younger adults **(B)**. *P ≤ 0.05, **P ≤ 0.01, ***P ≤ 0.001. No, no-attentional-load condition; Single, single-task-attentional-load condition; Dual, dual-attentional-load condition.

The RTs were shown for older adults and younger adults (Figure 2A). The 2_group_ (Older, Younger) × 3_load_ (No attentional load, Single-task attentional load, Dual-task attentional load) × 3_stimulus_ (A, V, AV) ANOVA for RTs showed a significant main effect of group [*F*_(1, 37)_ = 27.931, *P* < 0.001, η*_*p*_*^2^ = 0.430], with a faster response to the target by the younger adults than by the older adults, and an attentional load main effect [*F*_(2, 74)_ = 376.045, *P* < 0.001, η*_*P*_*^2^ = 0.910], with a faster response to the target for the single-task-attentional-load condition than for the dual-task- and no-attentional-load conditions (Single-task attentional load > No attentional load > Dual-task attentional load). There was also a significant main effect of stimulus type [*F*_(2, 74)_ = 101.302, *P* < 0.001, η*_*P*_*^2^ = 0.732], with a faster response to the bimodal AV target than to the individual auditory or visual targets (AV > A > V). Additionally, a significant interaction was found between group and attentional load [*F*_(2, 74)_ = 4.987, *P* = 0.021, η*_*P*_*^2^ = 0.119]. *Post hoc* analysis showed that for both older and younger groups, the response to the target was slower for the dual-task-attentional-load condition than for the no- (all *P* < 0.001) and single-task-attentional-load (all *P* < 0.001) conditions; however, there was no significant difference between the no- and single-task-attentional-load conditions (all *P* ≥ 0.578). The pairwise analysis of attentional load showed faster responses to targets for younger adults than for older adults for all attentional-load conditions (all *P* < 0.001). No other significant interactions were observed for RTs (all *P* ≥ 0.075).

The hit rate was also shown for older adults and younger adults (Figure 2B). The 2_group_ (Older, Younger) × 3_load_ (No attentional load, Single-task attentional load, Dual-task attentional load) × 3_stimulus_ (A, V, AV) ANOVA for hit rate showed a significant group main effect [*F*(1, 37) = 22.840, *P* < 0.001, η*_*P*_*^2^ = 0.382], with a higher hit rate for younger adults than for older adults, and a significant attentional load main effect [*F*_(2, 74)_ = 13.721, *P* < 0.001, η*_*P*_*^2^ = 0.271], with a lower hit rate in the dual-task-attentional-load condition (No attentional load = Single-task attentional load > Dual-task attentional load). There was also a significant main effect of stimulus type [*F*_(2, 74)_ = 10.359, *P* < 0.001, η*_*P*_*^2^ = 0.219], with a higher hit rate for the response to the AV target than to the A and V targets (AV > A > V). Additionally, a significant interaction was found between group and stimulus [*F*_(2, 74)_ = 11.614, *P* < 0.001, η*_*P*_*^2^ = 0.239]. *Post hoc* analysis showed that for older adults, the hit rate for V was lower than that for A (*P* < 0.001) and AV (*P* < 0.001), but there was no significant difference between A and AV (*P* = 0.309). For younger adults, no significant difference was found among A, V, and AV (all *P* = 1.000). *Post hoc* analysis for attentional load revealed a significant difference between older and younger adults for all attentional-load conditions (all *P* ≤ 0.016). No other significant interactions were found for hit rate (all *P* ≥ 0.053).

### Audio-Visual Integration

**FIGURE 3:**
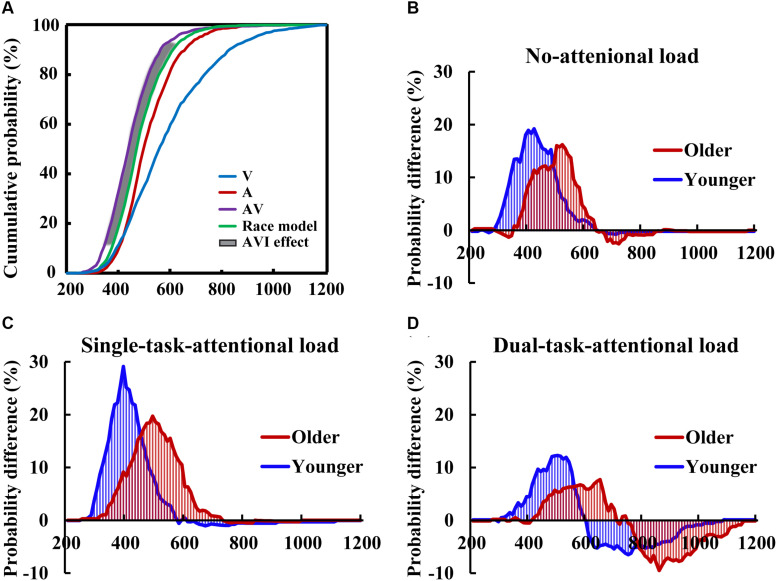
CDFs for the response times to the auditory, visual, audio-visual stimuli, and race models for the younger adults in the single-task-attentional-load condition **(A)** The race model analysis showed higher AVI effect for younger adults than that for older adults in all of the no- **(B)**, single-task- **(C)**, and dual-task-attentional-load **(D)** conditions.

Two-tailed *t-tests* were conducted between the CDFs of AV and the race model to evaluate the AVI effect in each 10-ms time bin for each group for each condition (Figure 3A). The results showed that AVI occurred for all conditions (all *P* < 0.05), and it was greatest for the single-task-attentional-load condition in both older and younger adults (Figures 3B–D), indicating that AVI was greatly influenced by attentional load, and the modulation of attentional load on AVI effect was different between single- and dual-tasks. In addition, the older adults showed a lower AVI effect than the younger adults in all attentional load conditions, exhibiting 16.01 vs. 19.21%, 18.34 vs. 29.13%, and 8.92 vs. 12.29% for the no-, single- task-, and dual-task-attentional-load conditions, respectively (Table 1). Additionally, the older adults showed a delayed AVI effect compared to that of younger adults, exhibiting delayed peak latency of 520 vs. 420 ms, 510 vs. 390 ms, and 650 vs. 500 ms for the no-, single- task-, and dual-task-attentional-load conditions, respectively, and a delayed time window for AVI of 420–610 vs. 310–510 ms, 340–630 vs. 290–490 ms, and 430–650 vs. 350–560 ms for the no-, single- task-, and dual-task-attentional-load conditions, respectively (Table 1).

**TABLE 1:** Peak benefit, peak latency, and time window of AVI for each attentional-load conditions.

	Peak benefit (%)		Peak latency (ms)		Time window (ms)	
	Older	Younger	Older	Younger	Older	Younger
No-attentional load	16.01	19.21	520	420	420–610	310–530
Single-task-attentional load	18.34	29.13	510	390	340–630	290–490
Dual-task-attentional load	8.92	12.29	650	500	430–650	350–560

### Theta/Alpha Activity and Audio-Visual Integration

**FIGURE 4:**
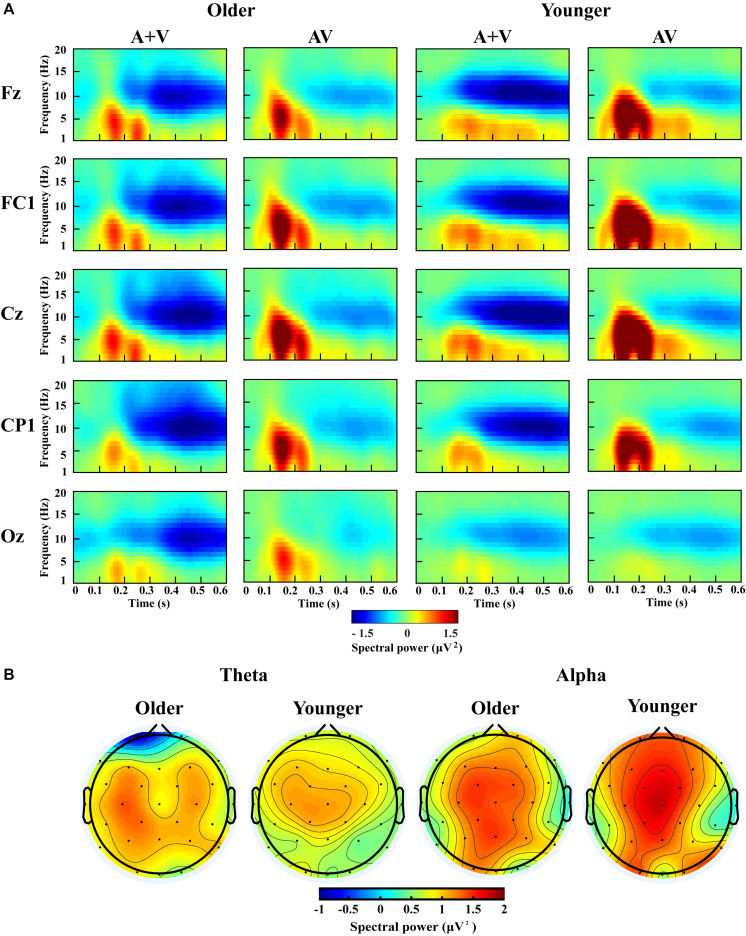
Spectral power activity of audio-visual (AV) and summed auditory and visual (A + V) data for a time interval of 0–600 ms in the representative electrodes **(A)** and topographic maps for the theta (100–400 ms) and alpha (200–600 ms) activity of audio-visual (AV) minus summed auditory and visual (A + V) data in the no-attentional-load condition **(B)**.

**FIGURE 5:**
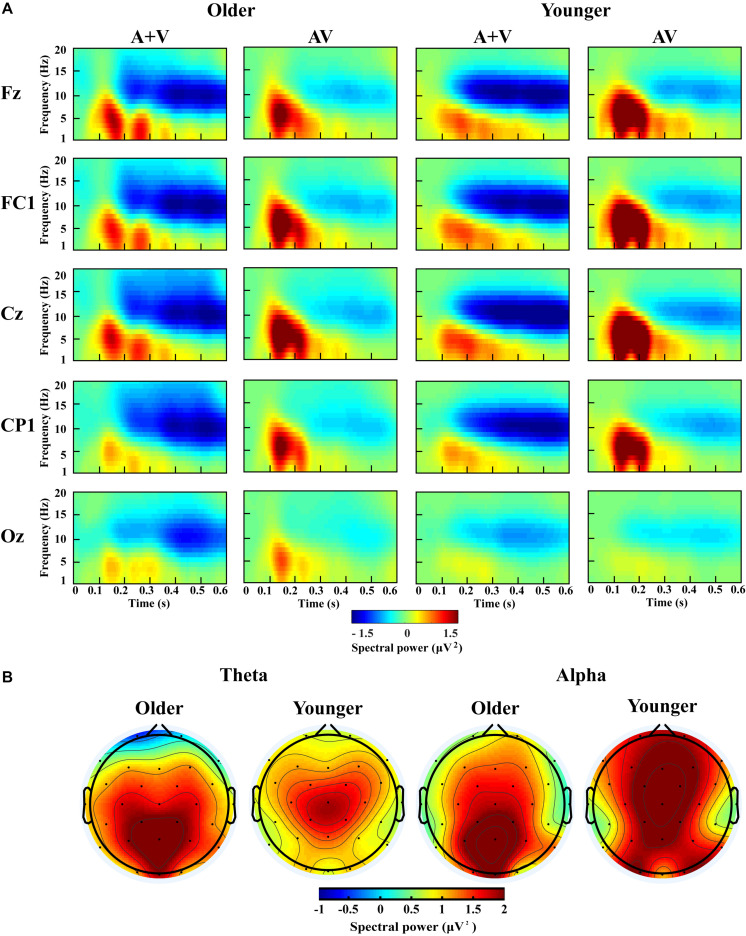
Spectral power activity of audio-visual (AV) and summed auditory and visual (A + V) data for a time interval of 0–600 ms in the representative electrodes **(A)** and topographic maps for the theta (100–400 ms) and alpha (200–600 ms) activity of audio-visual (AV) minus summed auditory and visual (A + V) data in the single-task-attentional-load condition **(B)**.

**FIGURE 6:**
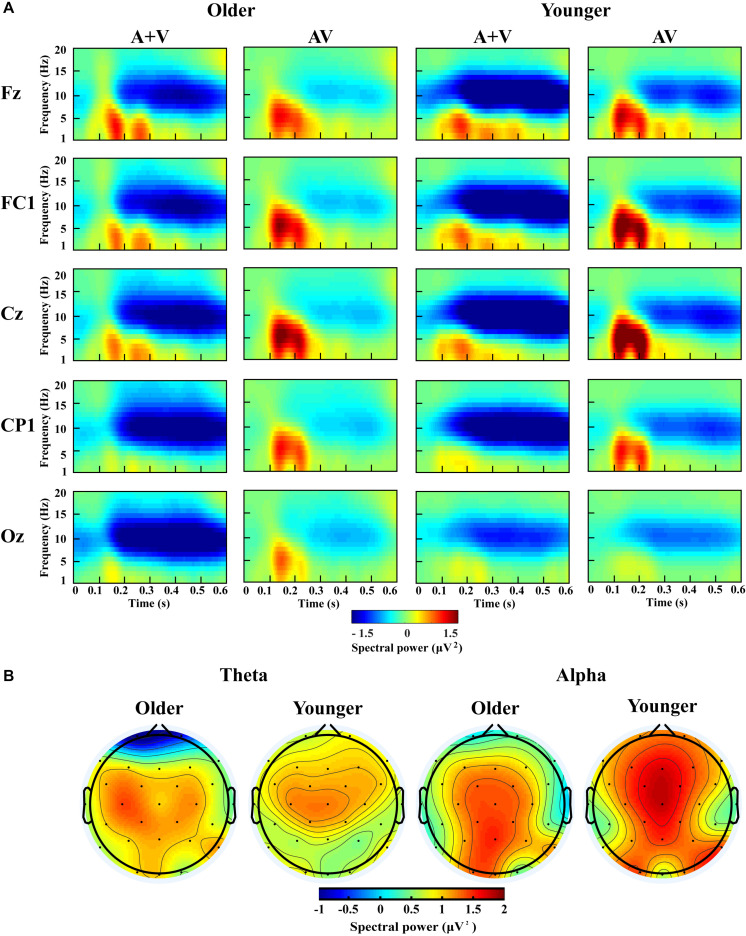
Spectral power activity of audio-visual (AV) and summed auditory and visual (A + V) data for a time interval of 0–600 ms in the representative electrodes **(A)** and topographic maps for the theta (100–400 ms) and alpha (200–600 ms) activity of audio-visual (AV) minus summed auditory and visual (A + V) data in the dual-task-attentional-load condition **(B)**.

According to previous studies and topographic response patterns, five regions of interest (ROIs) (frontal: F7, F3, Fz, F4, F8; fronto-central: FC5, FC1, FC2, FC6; central: C3, Cz, C4; centro-parietal: CP5, CP1, CP2, CP6; and occipital: O1, Oz, O2) in the 0–600 ms time interval were selected (Yang et al., 2015; Ren et al., 2018b). One-way ANOVA showed no significant lateralization effect for any of these ROIs; therefore, we chose one representative electrode with the highest activity in each ROI (Fz, FC1, Cz, CP1, and Oz) for further analysis. The grand-averaged event-related potentials for AV and (A + V) and topography maps for [AV–(A + V)] was showed in Supplementary Material. The transformed oscillatory response to AV trials and the transformed oscillatory response of summed A and V trials (A + V) were obtained, as shown in Figure 4A for the no-attentional-load condition, Figure 5A for the single-task-attentional-load condition, and Figure 6A for the dual-task-attentional-load condition. To establish the existence of oscillatory AVI effect, the two-tailed *t-test* comparison between the transformed oscillatory AV and (A + V) was performed across the 5 representative electrodes. Significant difference was found in the time interval of 100–400 ms for theta responses and 200–600 ms for alpha responses, as Figures 4B, 5B, 6B. Then the overall average spectral power across the selected time interval 100–400 ms for theta (4–7 Hz) and 200–600 ms for alpha (8–13 Hz) frequencies was submitted to 2_group_ (Older, Younger) × 3_load_ (No attentional load, Single-task attentional load, Dual-task attentional load) × 5_electrode_ (Fz, FC1, Cz, CP1, Oz) × 2_stimulus_ (AV, A + V) ANOVA to investigate the effect of attentional load on AVI and its aging effect.

For theta power, there was a significant main effect of load [*F*_(2, 76)_ = 7.098, *P* = 0.002, η*_*P*_*^2^ = 0.157], with higher theta activity in the no- and single-task-attentional-load conditions (No attentional load = Single-task attentional load > Dual-task attentional load), and a main effect of stimulus [*F*_(1, 38)_ = 4.683, *P* = 0.037, η*_*P*_*^2^ = 0.110], showing higher theta activity for AV than the sum of (A + V). This indicated that significant AVI occurred. A significant interaction between attentional load and stimulus [*F*_(2, 76)_ = 3.257, *P* = 0.050, η*_*P*_*^2^ = 0.079] was found. *Post hoc* analysis illustrated that significant AVI occurred in all attentional load conditions [AV > (A + V), all *P* ≤ 0.027]. However, theta activity was higher in the single-task-attentional-load than in the no- and dual-task-attentional-load conditions (Single-task attentional load > No-attentional load > Dual-task attentional load) for AV, and no significant difference was found between the single-task- and no-attentional load conditions in (A + V) (Single-task attentional load = No-attentional load > Dual-task attentional load), indicating that the AVI effect was higher in the single-task-attentional-load condition than in the no- and dual-task-attentional-load conditions. Additionally, an interaction of group × load × electrode [*F*_(8, 304)_ = 4.225, *P* = 0.012, η*_*P*_*^2^ = 0.100] was found. *Post hoc* analysis showed that for older adults, there was no significant difference among the no-, single- task-, and dual-task-attentional-load conditions at Fz and FC1, but there was significantly higher theta activity for the single-task-attentional-load condition than for the no- and dual-task-attentional-load conditions at Cz, CP1, and Oz (Single-task attentional load > No attentional load = Dual-task attentional load). Besides, there are significant differences between AV and (A + V) at Cz, CP1, and Oz (all *P* ≤ 0.038), but not at Fz and FC1 (all *P* ≥ 0.064). For younger adults, there was no significant difference among the no-, single- task-, and dual-task-attentional-load conditions in CP1 and Oz, but there was significantly higher theta activity for single-task-attentional-load conditions at Fz, FC1, and Cz (Single-task attentional load > No attentional load = Dual-task attentional load). Besides, there are significant differences between AV and (A + V) at Fz, FC1, and Cz (all *P* ≤ 0.041), but not at CP1 and Oz (all *P* ≥ 0.249). These results illustrated that the AVI oscillatory activity was the highest in the single-task-attentional-load condition in both older and younger adults, however, it mainly occurred at Cz, CP1, and Oz for older adults and at Fz, FC1, and Cz for younger adults. No other main effects and interaction were observed (all *P* ≥ 0.12).

For alpha activity, there was a significant main effect of attentional load [*F*_(2, 76)_ = 5.009, *P* = 0.014, η*_*P*_*^2^ = 0.116], showing higher theta activity in the no- and single-task-attentional-load conditions (No-attentional load = Single-task-attentional load > Dual-task-attentional load), and a main effect of stimulus [*F*_(1, 38)_ = 11.082, *P* = 0.002, η*_*P*_*^2^ = 0.226], showing higher theta activity for AV than the sum of (A + V). A significant interaction between attentional load and stimulus [*F*_(2, 76)_ = 3.539, *P* = 0.041, η*_*P*_*^2^ = 0.085] was found. *Post hoc* analysis illustrated that significant AVI occurred in all attentional load conditions [AV > (A + V), all *P* ≤ 0.005]. However, alpha activity was higher in the single-task- and no-attentional-load conditions than in the dual-task-attentional-load condition (Single-task-attentional load = No-attentional load > Dual-task-attentional load) for (A + V), and no significant difference was found among the three attentional load conditions in AV (Single-task-attentional load = No-attentional load = Dual-task-attentional load), indicating a higher AVI effect for the single-task-attentional load condition than for the no- and dual-task-attentional-load conditions. No other main effects and interactions were observed (all *P* ≥ 0.09).

### Theta/Alpha and MRE

**FIGURE 7:**
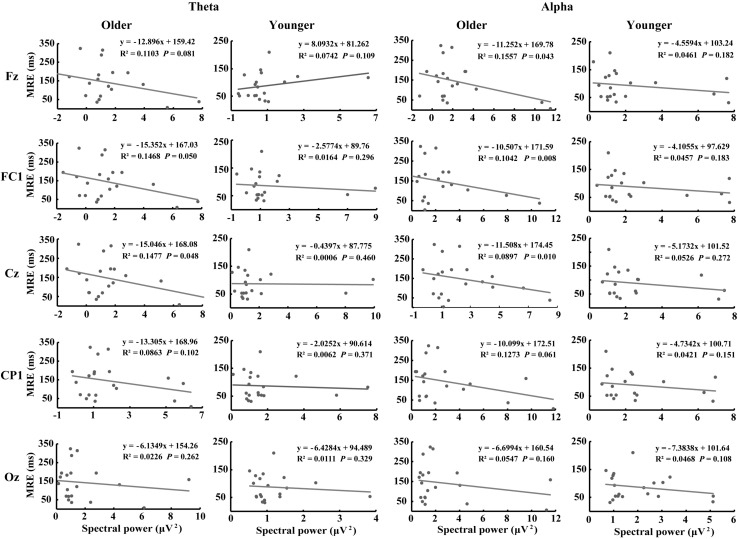
Correlations between evoked theta- and alpha-AVI oscillatory responses and MRE in the dual-task-attentional-load condition for the representative electrode in each ROI.

Figure 7 shows the correlations between MRE and theta- and alpha-AVI oscillatory responses at the 5 electrodes in the dual-task-attentional-load condition. The correlation in the no- and single-task-attentional-load conditions was not significant; therefore, these correlations are not shown or discussed further. Significant negative correlations between the MRE and FC1 (*r*^2^ = 0.1468, *P* = 0.05) and Cz (*r*^2^ = 0.1447, *P* = 0.048) were observed for theta-AVI oscillatory response. In addition, significant negative correlations between MRE and Fz (*r*^2^ = 0.1557, *P* = 0.043), FC1 (*r*^2^ = 0.1042, *P* = 0.008) and Cz (*r*^2^ = 0.0897, *P* = 0.010) alpha-AVI oscillatory responses were found. These findings illustrated that the AVI effect decreased as attentional load increased in the dual-task-attentional-load condition, which suggested a negative correlation between AVI effect and attentional load in dual task. No other significant correlations occurred (all *P* > 0.24).

## Discussion

Our study aimed to investigate the effect of attentional load on AVI and its aging effect in single- and dual-tasks. The results showed that the highest AVI effect was found for the single-task-attentional-load condition in both older and younger adults, and the AVI was weaker and delayed in older adults. AVI oscillatory activity mainly occurred in the Cz, CP1, and Oz of older adults but in the Fz, FC1, and Cz of younger adults. In addition, significant negative correlations between behavioral MRE and AVI oscillatory activity were found for the older adults but not for the younger adults in the dual task.

Consistent with our original hypothesis that the attentional load affect AVI regardless of attentional-load conditions, but modulation of attentional load on AVI effect was different when the attentional load was from single-task and dual-task. Both the behavioral and EEG oscillatory results illustrated that the AVI effect was higher in the single-task-attentional-load condition than in the no- and dual-task-attentional-load conditions in both older and younger adults. Theta oscillation has been suggested to be associated with attention arousal (Keller et al., 2017), and activity in the alpha band has been suggested to be associated with the suppression of distracting signals (Yordanova et al., 1998; Friese et al., 2016; Keller et al., 2017). In the present study, the theta and alpha oscillations were higher in the single-task-attentional-load condition than in the no- and dual-task-attentional-load conditions for both older and younger adults, which illustrated that the peripheral target captured many more attentional resources under the single-task-attentional-load condition. Compared with the no-attentional-load condition, more attentional resources were activated (Lavie and Tsal, 1994), given that attention can positively facilitate AVI (Talsma et al., 2007; Talsma et al., 2009, 2010); therefore, the AVI effect was greater in the single-task-attentional-load condition than in the no-attentional-load condition. However, in the dual-task-attentional-load condition, the participants were instructed to response to the additional distractors in the RSVP task simultaneously, which lead to that the attentional resources were largely occupied by RSVP task, exhibiting a significantly lower hit rate. Therefore, more attentional resources were diverted to the competing RSVP task, which led to insufficient attentional resources to complete the AV discrimination task simultaneously, especially for older adults (Lavie and Tsal, 1994). Younger adults could complete the dual task with less difficulty, showing a much higher hit rate than older adults, however, the hit rate was significantly lower than that under the no-attentional-load and single-task-attentional-load conditions. Therefore, the attentional load of younger individuals in the dual-task-attentional-load condition was almost full, and there were fewer attentional resources left to process peripheral stimuli than in the single-task-attentional-load condition. Therefore, the AVI effect was significantly reduced in the dual-task-attentional-load condition compared with the single-task-attentional-load and no-attentional-load conditions.

In addition, in all conditions, the AVI effect of older adults was weaker and delayed than younger adults, which was consistent with findings in previous researches (Mahoney et al., 2011; Wu et al., 2012; Ren et al., 2016). However, controversial findings have been extensively reported (Laurienti et al., 2006; Peiffer et al., 2007; Diederich et al., 2008). Attention facilitates the AVI effect in multiple stages (Talsma and Woldorff, 2005; Talsma et al., 2007, 2009, 2010), however, a great deal of studies found the attentional deficits in older adults (Fraser and Bherer, 2013; Williams et al., 2016). Therefore, attention might be an important factor contributing to the weaker AVI effect in older adults. Additionally, in the studies in which the older adult exhibited relatively higher AVI effect than younger adults, the auditory and visual stimuli were presented centrally (Laurienti et al., 2006; Peiffer et al., 2007; Diederich et al., 2008), however, in the current study, the auditory and visual stimuli were presented on the upper/lower left or right quadrant of a screen with a 12-degree visual angle peripherally. Investigations have illustrated that peripheral resolution declines with age (Anderson and Mcdowell, 1998); therefore, another possible reason for the conflicting conclusions might be the location of the stimuli presented. The delayed AVI effect in older adults compared to that of younger adults was consistent with the findings of numerous previous studies, such as Wu et al. (2012), Ren et al. (2016), and Wang et al. (2017, 2018). Colonius et al. proposed a “*time-window-of-integration model*,” which proposing that the integration of audiovisual integration includes at least two serial stages: an early afferent stage of peripheral processing (first stage) and a compound stage of converging sub-processes (second stage) (Colonius and Diederich, 2004; Diederich et al., 2008). In the first stage, the processing of the unimodal sensory information was regarded as independent. If both of the unimodal visual information and unimodal auditory information in the first stage terminate within a given time course, the AVI is assumed to occur in the second stage (Xu et al., 2020). Comparing to the younger adults, the older adults exhibited a relatively higher perceptual threshold for auditory and visual stimuli and a slower processing speed in the first stage (Spear, 1993; Liu and Yan, 2007), which led to a delay for the second stage. Therefore, the delayed AVI might be mainly due to unimodal functional decline.

Considering that no significant lateralization effect for any of all ROIs, and the representative electrodes were used to conduct the AVI oscillation analysis. The results showed that the significant AVI effect mainly occurred in the Cz, CP1, and Oz for older adults but in the Fz, FC1, and Cz for younger adults. These results suggest that the AVI effect might be shifts from the anterior region to the posterior region with age, which the current study is the first to report. Recently, Ren et al. (2018b) investigated age-related AVI using ERPs. Their results showed that significant AVI was elicited in an established visual processing brain region (the occipital cortex) in older adults but not in younger adults, which indicated that the compensatory mechanism for reduced neural function occurred. Their audio-visual spatiotemporal perceptual training experiment in healthy older adults showed robust malleability of the aging brain (Yang et al., 2018). Additionally, Wang et al. examined aging adaption from the view of functional connectivity during AV processing in older adults using an AV discrimination task, and their results showed that older adults activated stronger connections than younger adults (Wang et al., 2017, 2018). Furthermore, a similar shift adaptive mechanism during memory tasks in the aging brain has been found (Davis et al., 2007). Therefore, we propose that as a supporting framework, older adults activate different brain networks to integrate auditory and visual stimuli than younger adults do, exhibiting the anterior-to-posterior shift of AVI processing. However, the present EEG study is difficult to provide the previous source location information, and the further fMRI studies are needed.

Additionally, a negative correlation between AVI effect and attentional load was found for older adults in the dual task but not for younger adults, which is also reported for the first time in this study. In the dual task, the participant was asked to treat the peripheral discrimination task and central RSVP task equally. Therefore, more attentional recourse was occupied by distractors of RSVP task, fewer attention resources were left to process stimuli during the AV discrimination (Lavie and Tsal, 1994). Previous studies found that the AVI effect was higher in the attended location than in the unattended location (Talsma and Woldorff, 2005; Talsma et al., 2007, 2009, 2010). Therefore, it is reasonable that the AVI effect decreased with an increasing attentional load for older adults. Compared with the aging brain, the younger brain is more flexible and efficient (Grady, 2012), showing higher executive function (Baum et al., 2017). When performing the dual task, younger adults’ attentional resources were sufficient to process all stimuli; their hit rate for all stimuli was greater than 95%. In addition, the current results showed that older adults exhibited the same brain oscillation in all attentional load conditions. However, younger adults exhibited similar oscillatory patterns to older adults in the dual-task condition and exhibited unique oscillations in the single-task conditions. Therefore, it is reasonable that no significant negative correlation was found between the AVI effect and attentional load for younger adults.

In conclusion, the attentional load affect AVI greatly, and the modulation of attentional load on AVI effect was different when the attentional load was from single-task and dual-task. In addition, the AVI effect for peripheral stimuli was delayed and weaker in older adults compared to younger adults, but as an adaptive mechanism of the aging brain, the AVI oscillation might be shifted from the anterior to the posterior regions in older adults under all attentional loads. However, as a limitation that only a visual distractor was used in the current study, the different results might be obtained if the additional distractor was from auditory modality. Therefore, further studies that manipulates attentional recourse through auditory distractors were needed.

## Data Availability Statement

The raw data supporting the conclusions of this article will be made available by the authors, without undue reservation.

## Ethics Statement

The studies involving human participants were reviewed and approved by the Ethics Committee of Hubei University and the Second Affiliated Hospital of Guizhou University of Traditional Chinese Medicine. The patients/participants provided their written informed consent to participate in this study.

## Author Contributions

YR and WY conceived and designed the experiments. SL and TW collected the data. YR analyzed the data, wrote the draft manuscript, and received the comments from WY. All authors contributed to the article and approved the submitted version.

## Conflict of Interest

The authors declare that the research was conducted in the absence of any commercial or financial relationships that could be construed as a potential conflict of interest.

## Footnotes

1 http://sccn.ucsd.edu/eeglab/
