# Supplementary material for: Age-Related Shifts in Theta Oscillatory Activity During Audio-Visual Integration Regardless of Visual Attentional Load
Source: Front Aging Neurosci. 2020 Sep 30;12:571950. doi: 10.3389/fnagi.2020.571950 (PMC7556010; doi:10.3389/fnagi.2020.571950)
Supplement: Supplementary file 1 [file Data_Sheet_1.PDF]

Supplementary Material

According to previous studies, five regions of interests (ROIs) (frontal: F7, F3, Fz, F4, F8; fronto-central: FC5, FC1, FC2, FC6; central: C3, Cz, C4; centro-parietal: CP5, CP1, CP2, CP6; and occipital: O1, Oz, O2) in the 0 – 600 ms time interval and the 1 – 40 Hz frequency range were selected. According to the one-way ANOVA for each ROI, there was no significant lateralization effect; therefore, we chose one electrode in each ROI (Fz, FC1, Cz, CP1 and Oz). the Grand-averaged event-related potentials and Topography maps were showed in **Supplementary Figure 1** for no-attentional-load condition, **Supplementary Figure 2** for single-task-attentional-load condition, and **Supplementary Figure 3** for dual-task-attentional-load condition.

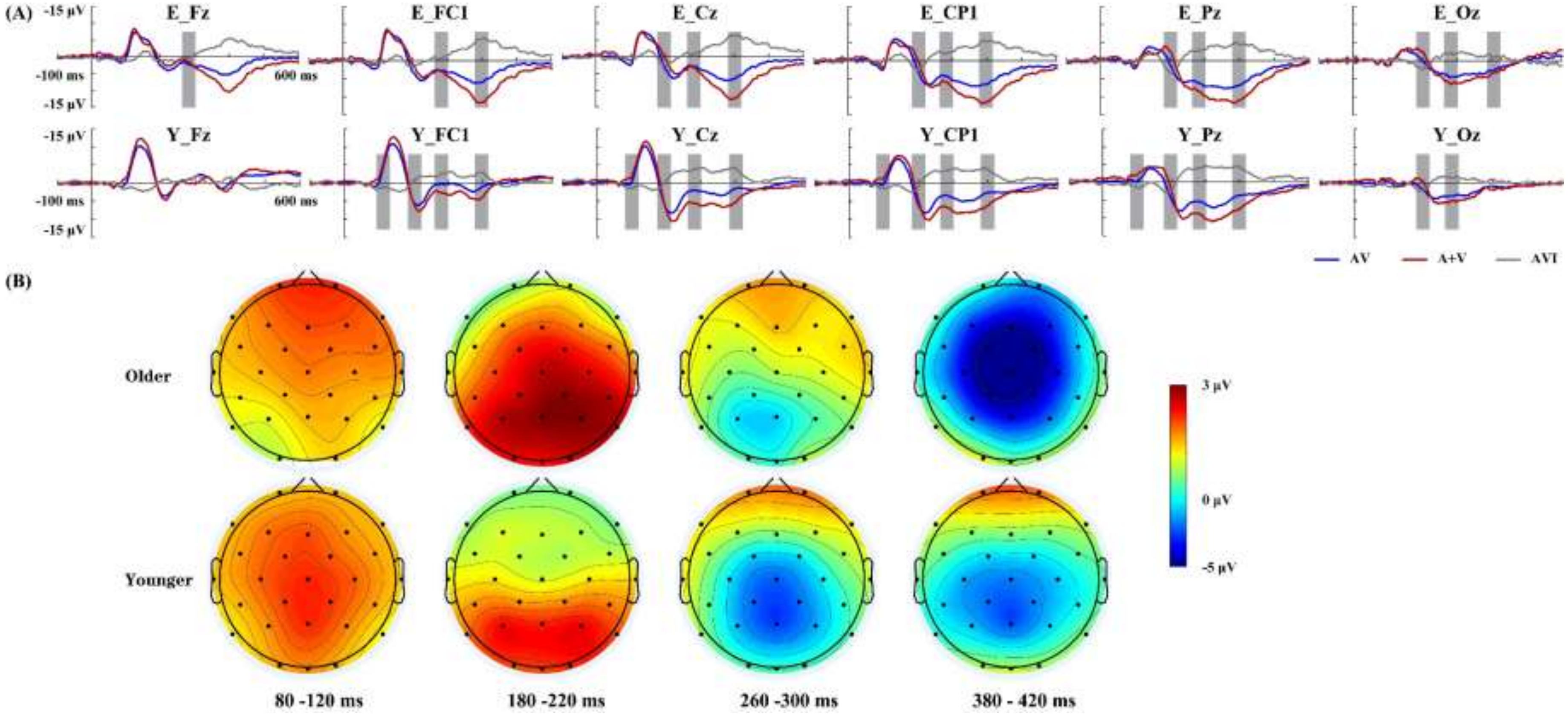

Supplementary Figure 1. Grand-averaged event-related potentials for AV and (A + V) (A) and Topography maps for [AV – (A + V)] (B) in no-attentional-load condition. The significant difference between AV and (A + V) was marked in grey background.

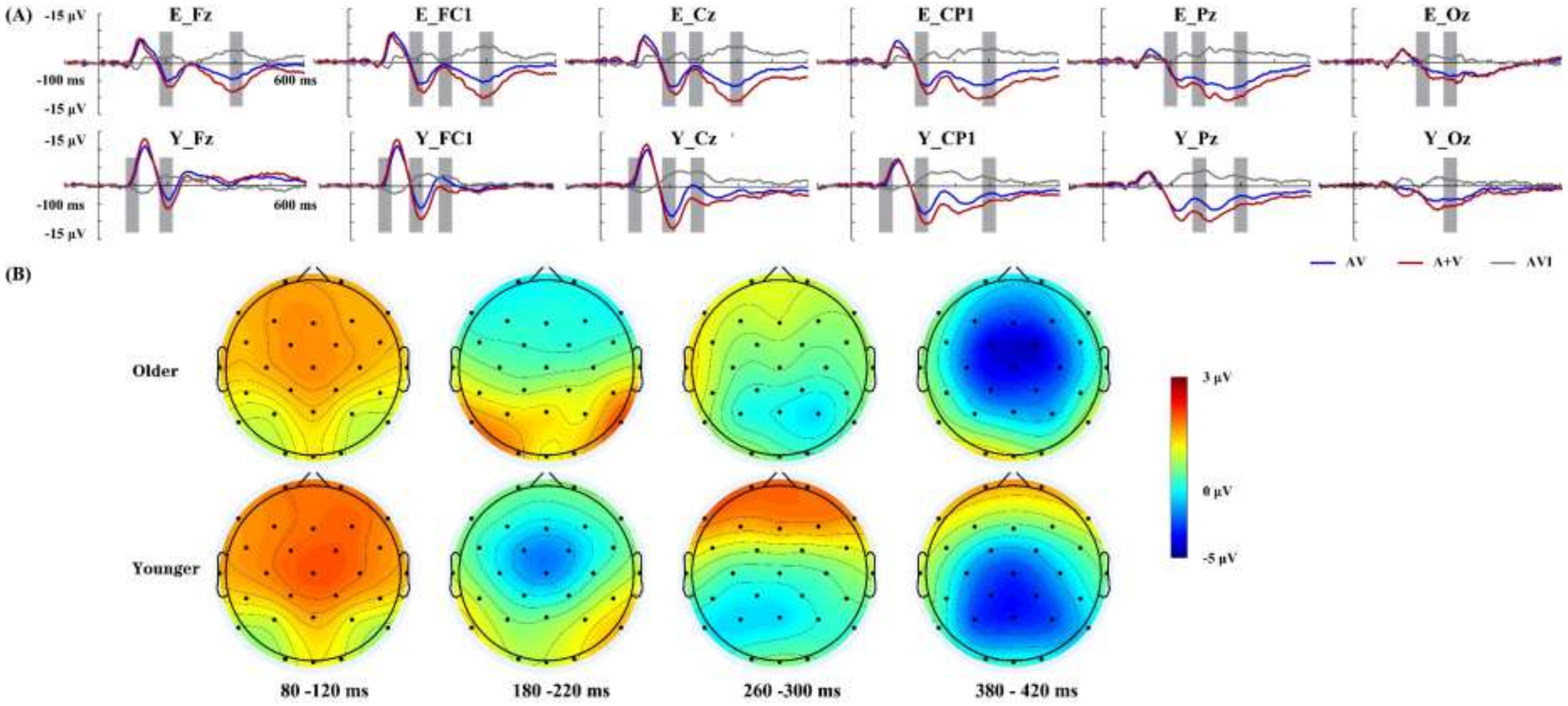

Supplementary Figure 2. Grand-averaged event-related potentials for AV and (A + V) (A) and Topography maps for [AV – (A + V)] (B) in single-task-attentional-load condition. The significant difference between AV and (A + V) was marked in grey background.

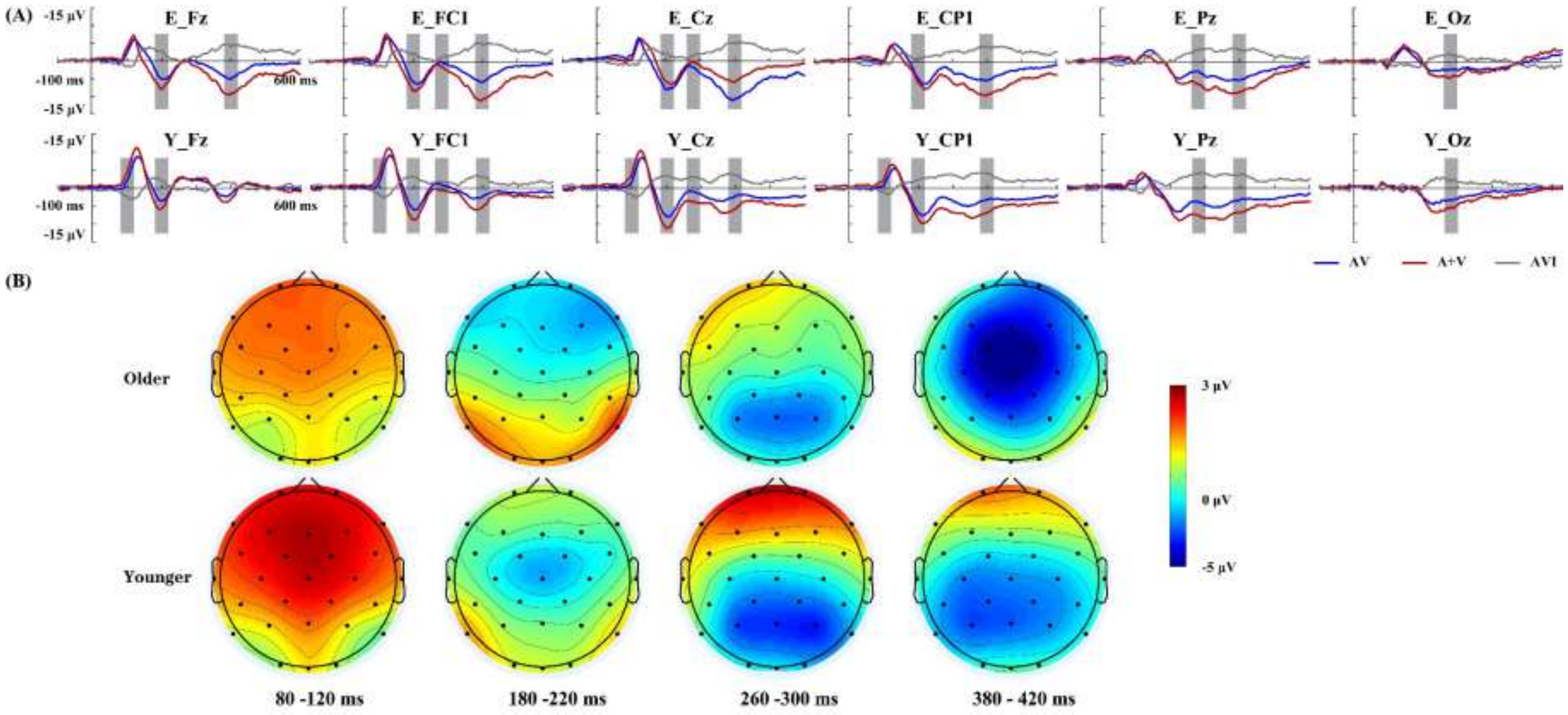

Supplementary Figure 3. Grand-averaged event-related potentials for AV and (A + V) (A) and Topography maps for [AV – (A + V)] (B) in dual-task-attentional-load condition. The significant difference between AV and (A + V) was marked in grey background.
